# Total Hip Arthroplasty Using a Short-Stemmed Femoral Component in the Presence of a Long Dynamic Condylar Screw Osteosynthesis Plate

**DOI:** 10.1155/2014/725403

**Published:** 2014-09-30

**Authors:** Martin Buttaro, Nicolas Piuzzi, Fernando Comba, Gerardo Zanotti, Francisco Piccaluga

**Affiliations:** The Hip Surgery Unit, Institute of Orthopaedics “Carlos E. Ottolenghi”, Italian Hospital of Buenos Aires, Potosí 4247, C1199ACK Buenos Aires, Argentina

## Abstract

We present a potential indication of a short-stemmed femoral component in a patient with multiple comorbidities presenting with hip posttraumatic osteoarthritis and a long dynamic condylar screw osteosynthesis plate. Removal of the plate and implantation of a long stem would have been related to a much longer operative time and potential local or systemic complications.

## 1. Introduction 

Although the origins of short-stemmed femoral components were in the 70s, nowadays these implants have become a more common indication especially in young patients, with the purpose of preserving proximal femoral bone. However, short-stemmed femoral components may be useful for atypical cases in older patients with an adequate bone quality and the need for a total hip arthroplasty (THA).

## 2. Case Report

**Figure 1:**
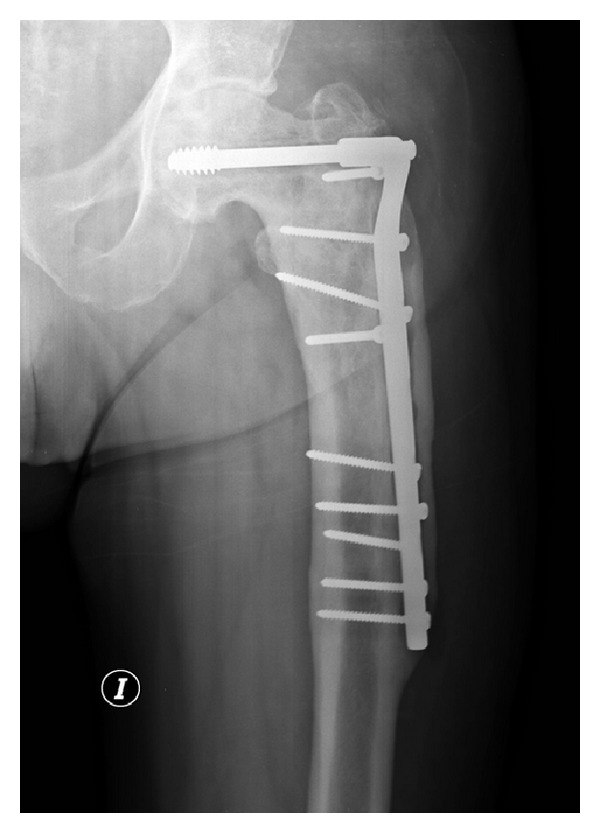
Preoperative AP radiograph of the left hip showing a healed intersubtrochanteric fracture with a dynamic condylar screw and osteosynthesis plate, coxa vara, and posttraumatic osteoarthritis.

A 72-year-old man presenting with groin pain irradiated to the thigh with a history of an intersubtrochanteric left hip fracture treated with a long dynamic condylar screw at another institution 8 years before being referred to us. Six years after the index operation the patient started with symptoms that were not able to respond to oral analgesics. Medical history included hypertension, coronary artery disease, cardiac insufficiency, and renal insufficiency without requiring dialysis. Functional preoperative score was 6 points according to the D'Aubigne and Postel score [1]. Radiographs showed fracture consolidation in 10° of varus in comparison with the anatomical femoral axis and 1.5 shortening of the left leg, with bone ingrowth along the distal 2/3 of the osteosynthesis plate (Figure 1). We indicated a short-stemmed prosthesis in order to avoid plate removal. Removal of previously fixed metallic devices would have necessitated an extensive soft-tissue dissection and a long operation time, which might have increased the risk of cardiac complications as well as infection. Preoperative assessment in our hospital indicated an ASA (American Society of Anesthesiologists) class 4 [2]. The patient was classified as a grade C (other severe comorbidities) according to Charnley's classification [3]. Radiographic preoperative templating using various femoral component designs confirmed that the cannon of the plate would jeopardize implantation of standard-length components. The operation was performed using epidural in lateral decubitus using a posterolateral approach. First step was removal of the 3 most proximal screws of the dynamic condylar screw plate. One of the screws was fractured during removal but the fractured portion remained inside the medial cortical of the femur without making any contact with the broach or the stem. Posterior dislocation was then performed and femoral neck cut was done with 4 sliced cuts to allow dynamic screw visualization.

**Figure 2:**
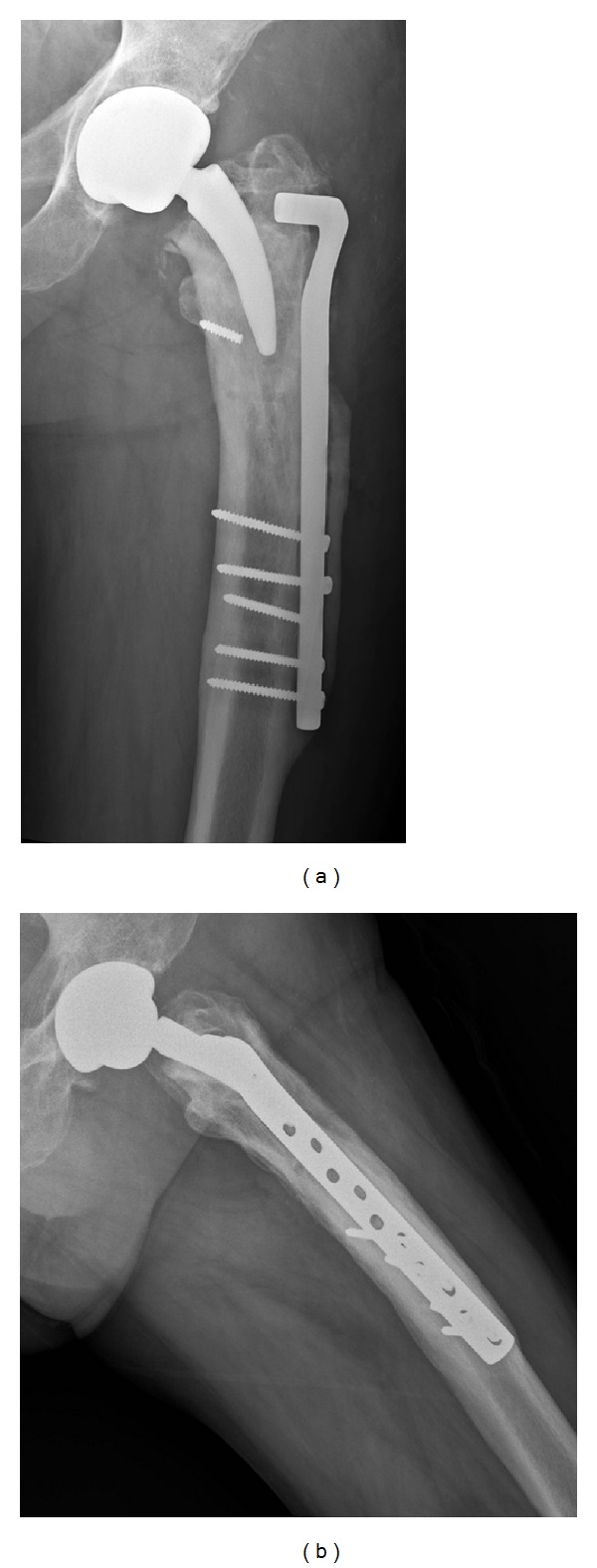
Anteroposterior (a) and lateral (b) radiographs of the left hip one year after the conversion to a short-stemmed THA.

Removal of this screw was performed in an anterograde way as it is not possible to take it away in a retrograde fashion without plate removal. The acetabular cup was first performed with the use of a 58 mm external diameter Trinity cup (Corin, Cirencester, UK) implanting a 40 mm ceramic liner (BIOLOX delta, CeramTec AG, Plochingen, Germany) in order to achieve the most possible stable hip in a patient with such comorbidities. Femoral broaching was performed with the use of an image intensifier in order to avoid cortical perforations. Trial reduction was performed trying to equalize limb lengthening. A number 2 MiniHip stem (Corin, Cirencester, UK) was implanted with a 40 mm ceramic femoral head (BIOLOX delta, CeramTec AG, Plochingen, Germany). Total operative time was 75 minutes. Postoperative radiographs showed no femoral perforations and equalization of limb length. The patient spent 24 hrs in intensive care unit and then started weight-bearing as tolerated with a walker during 2 weeks, followed by a 2-week period with a cane. No clinical complications were observed. Thromboembolic prophylaxis was continued until the 30th postoperative day using enoxaparin 40 mg daily. He was followed up at 45 and 90 days postoperative and then at 1 year, with radiographic signs of both femoral and acetabular bony ingrowth stability (Figures 2(a) and 2(b)) with a functional score of 14 points [1]. Unfortunately, the patient died in another clinic at 19 months postoperative due to a dissecant abdominal aorta aneurism. The family was requested on the hip function and the widow told us he was walking short distances with the use of a cane.

## 3. Discussion

Secondary hip osteoarthritis in patients with a previous fracture of the proximal femur is not an infrequent situation. Recommended steps in the case a THA needs to be performed are as follows: first, plate removal and, then, if the bone quality is adequate, total hip arthroplasty [4]. A long stem that bypasses the most distal screw hole is usually needed in this situation to avoid a femoral fracture through the most distal screw hole. In some cases, bone quality after hardware removal can be compromised, and THA may need to be deferred for a second stage. Furthermore, osteosynthesis plates that were implanted many years ago may be covered by bone or may have broken screws that may be technically difficult to be extracted. Resurfacing may be an option in some of these cases, but a metal on metal pairing couple would not be appropriate for a patient with such medical history. We preferred to use this short-stemmed prosthesis, which has been related to reconstruction of the individual geometry of the hip in 250 cases [5]. Short-stemmed femoral prosthesis has been related to 100% survivorship in patients with an average of 79 years old at a mean 4.6-year followup [6]. However, the massive use of these implants should be discouraged until prospective randomized studies demonstrating that conventional and short stems are related to similar results are published. This case highlights the importance of preoperative planning and the potential utility of unconventional stems to treat infrequent clinical situations.

## Supplementary Material

A 72 year-old man, ASA 4, presenting groin pain irradiated to the thigh with a history of an intersubtrochanteric left hip fracture treated at another institution 8 years before. Radiographs showed fracture consolidation in 10° of varus and 1.5 shortening of the left leg, with bone ingrowth along the distal 2/3 of the osteosynthesis plate. We indicated a short-stemmed prosthesis in order to avoid plate removal and decrease postoperative morbidity.
The operation was performed using epidural anaesthesia in a laminar flow room in lateral decubitus using a posterolateral approach. First step was removal of the three most proximal screws of the dynamic condylar screw plate. Then posterior dislocation was performed and femoral neck cut was done with 4 sliced cuts to allow dynamic screw visualization. Removal of this screw was performed in an anterograde way as it is not possible to take it away in a retrograde fashion without plate removal. The acetabular cup was first performed with the use of a 58 mm external diameter Trinity cup (Corin, Cirencester, UK) implanting a 40 mm ceramic liner (BIOLOX® delta, CeramTec AG, Plochingen, Germany) in order to achieve the most stable possible hip in a patient with such comorbidities. Femoral broaching was performed with the use of an image intensifier in order to avoid cortical perforations. Trial reduction was performed according to general rules and trying to equalize limb lengthening. A number 2 MiniHip stem (Corin, Cirencester, UK)) was implanted with a 40 mm ceramic femoral head (BIOLOX® delta, CeramTec AG, Plochingen, Germany) (Multimedia 1). Total operative time was 75 minutes. Postoperative radiographs showed no femoral perforations and equalization of limb length. The patient spent 24 hs in intensive care unit and then started weightbearing as tolerated with a walker during 2 weeks, followed by a 2 week period with a cane. No clinical complications were observed.
